# Supplementary figures and images for: Water chlorination increases the relative abundance of an antibiotic resistance marker in developing sourdough starters
Source: Microbiol Spectr. 2024 Sep 16;12(11):e01121-23. doi: 10.1128/spectrum.01121-23 (PMC11537093; doi:10.1128/spectrum.01121-23)

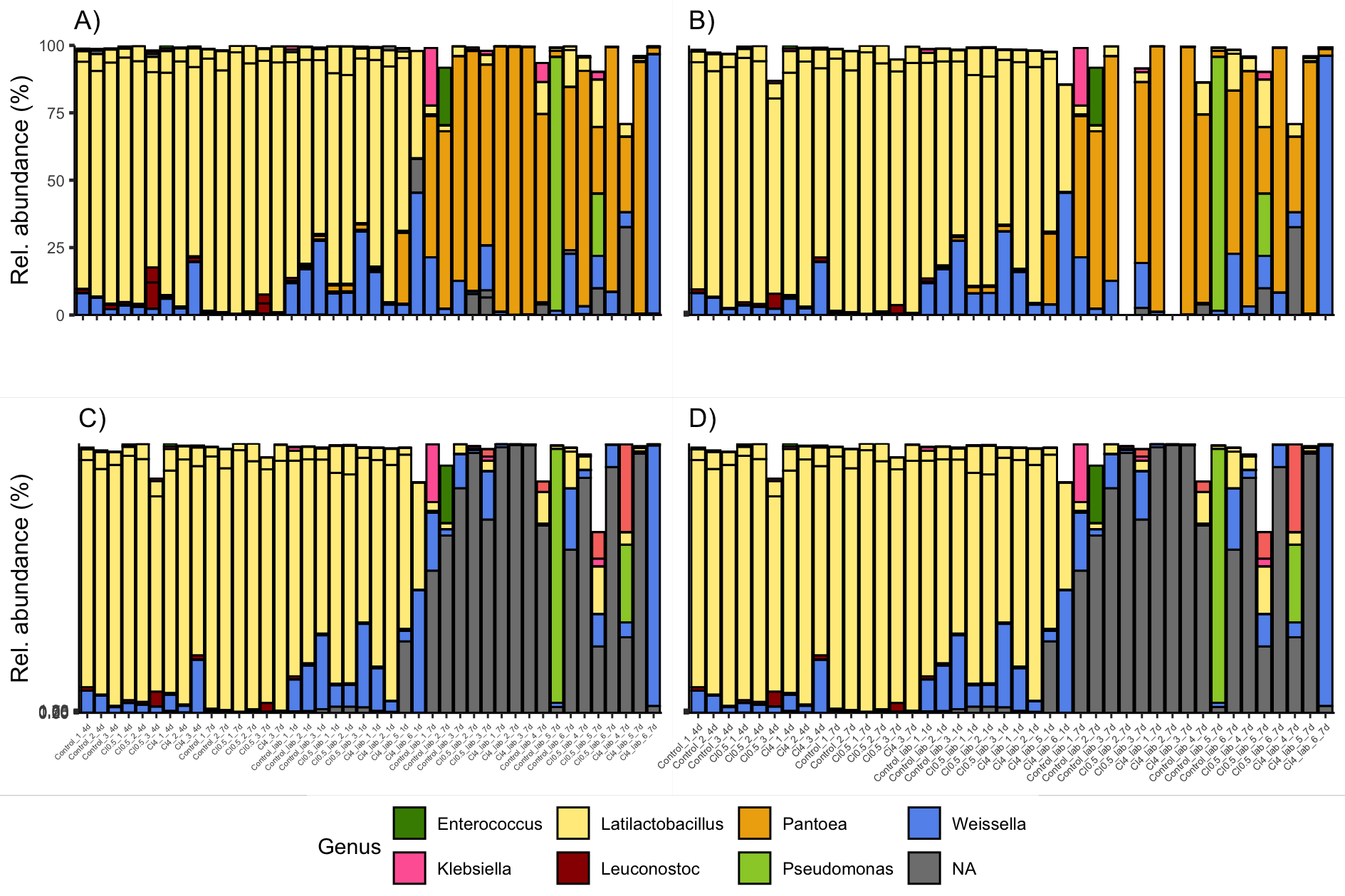

Supplement: Figure S1 — Comparing taxonomic assignment based on different databases. [file spectrum.01121-23-s0001.tif]

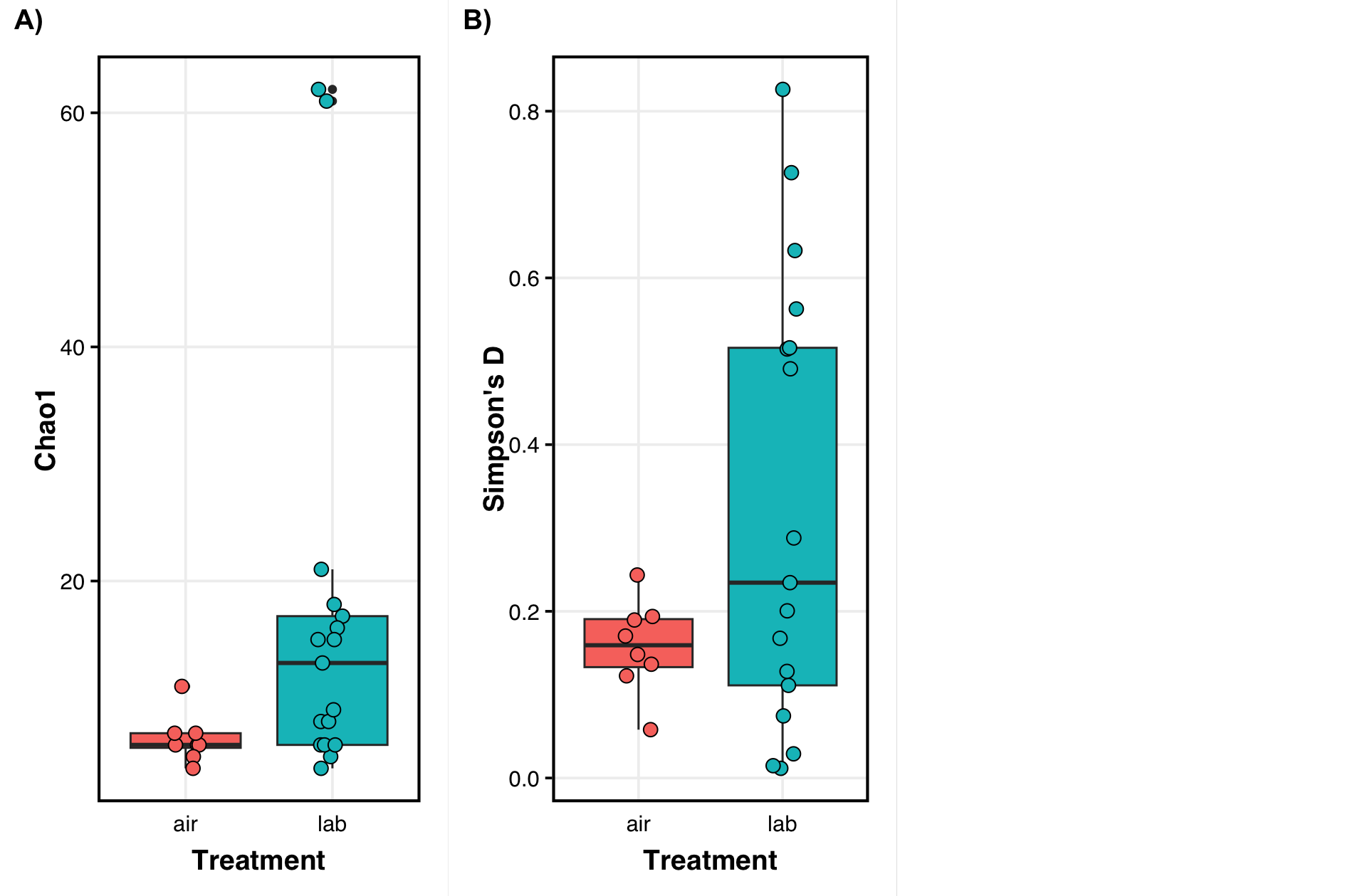

Supplement: Figure S2 — Effect of air exposure. [file spectrum.01121-23-s0002.tif]

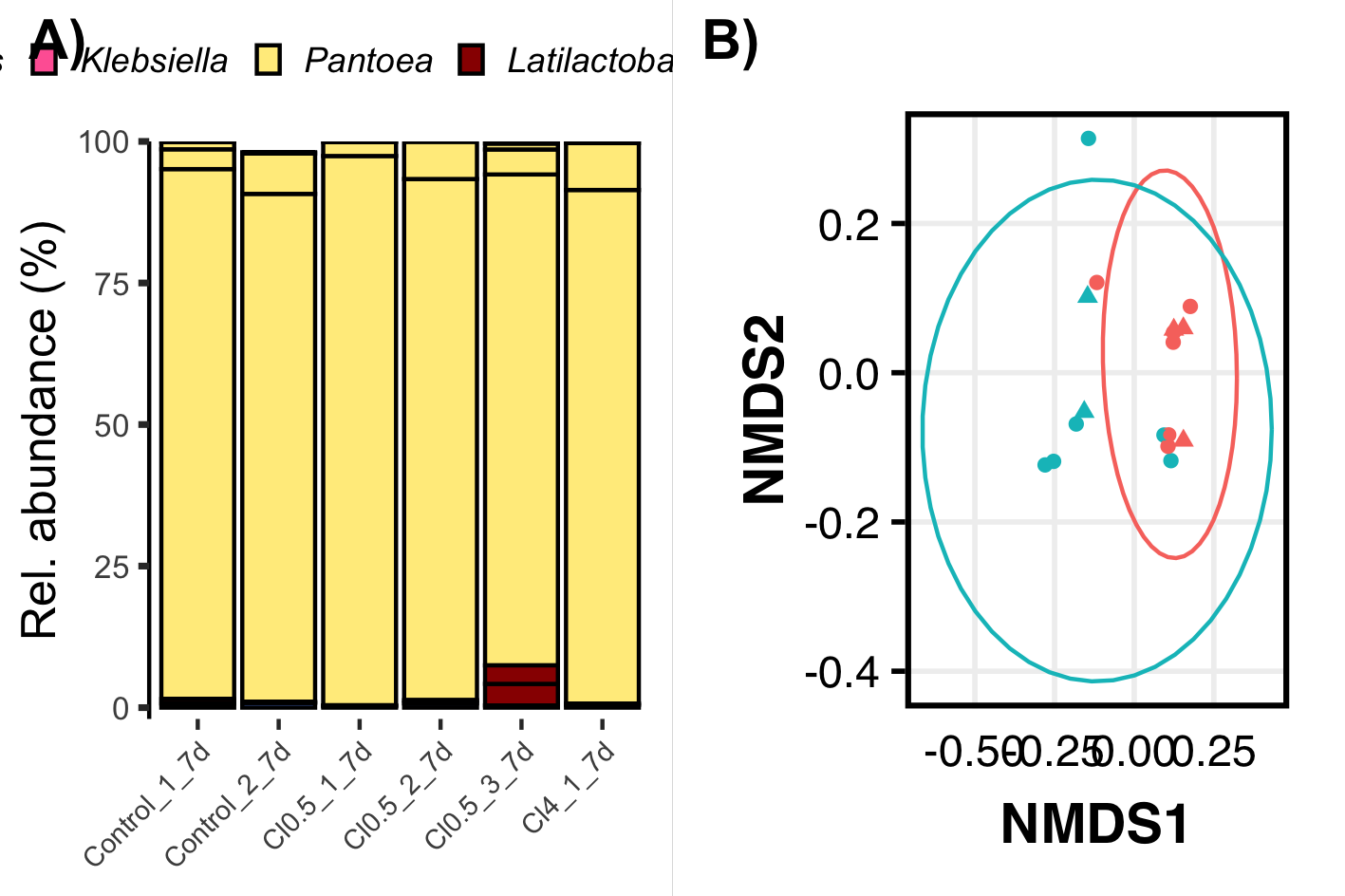

Supplement: Figure S3 — Effect of chlorination on bacterial community structure. [file spectrum.01121-23-s0003.tif]
