# Supplementary material for: Water chlorination increases the relative abundance of an antibiotic resistance marker in developing sourdough starters
Source: Microbiol Spectr. 2024 Sep 16;12(11):e01121-23. doi: 10.1128/spectrum.01121-23 (PMC11537093; doi:10.1128/spectrum.01121-23)
Supplement: Supplemental Figure Legend — Legends for Fig. S1, S2, and S3. [file spectrum.01121-23-s0004.docx]

**Supplementary Materials**

**Figure S1. The effect of database and algorithm choice on taxonomic identification of the most common bacteria variants**. Relative abundance of the top ten genera identified using the RDP training dataset v18 with a) the *DADA2* native “assignTaxonomy” function, and b) the IDTAXA function from the *DECIPHER* package as well as the SIVA training dataset v138.1 with the c) the *DADA2* “assignTaxonomy” function; and d) the IDTAXA function from *DECIPHER*. Genera are identified as follow: *Enterococcus* (chartreuse), *Klebsiella* (pink), *Latilactobacillus* (light goldenrod), *Leuconostoc* (plum), *Pantoea* (dark goldenrod), *Pseudomonas* (green), and *Weissella* (blue).

**Figure S2. Effect of exposure to unfiltered air on bacteria diversity indices. A)** Chao1, or the number of predicted ASVs (*F*_(1,23)_ = 2.83; adj-*P* = 0.22) and **B**) Diversity is measured as Simpson’s *D* (*F*_(1,23)_ = 3.01; adj-*P* = 0.20). Starters grown in the presence of unfiltered air are shown in red and starters grown in sterile laboratory conditions are shown in blue. Box plots show the median as well as the interquartile range. Individual points represent score for each sample.

**Figure S3. Effect of exposure to chlorination bacteria community structure**. (a) Relative abundance of the six most common bacteria genera: *Enterococcus* (green), *Klebsiella* (pink), *Pantoea* (corn), *Latilactobacillus* (dark gold), *Pseudomonas* (olive), *Erwinia* (cornflower blue), and unknown (Gray). (b) Differences in population structure estimated from Non-metric MultiDimensional Scaling of distance matrix calculated from the Bray-Curtis dissimilarity index. Bacteria types found in sourdough fermented in the presence of unfiltered and filtered air were more clustered than expected by chance alone. Sourdough starters fermented in fileted air are shown in blue, while starters fermented in unfiltered air are presented in red.
